# Relationship between hemoglobin levels and diabetic retinopathy in Chinese type 2 diabetes mellitus populations: a cross-sectional study

**DOI:** 10.3389/fendo.2026.1800238

**Published:** 2026-05-14

**Authors:** Jinghan Zheng, Jingwei Chi, Kui Che, Qidong Zheng, Yangang Wang

**Affiliations:** 1 Department of Endocrinology, Affiliated Hospital of Qingdao University, Qingdao, Shandong, China; 2 Department of Internal Medicine, Yuhuan Second People’s Hospital, Yuhuan, Zhejiang, China

**Keywords:** cross-sectional study, diabetic retinopathy, hemoglobin, sex‐stratified, type 2 diabetes

## Abstract

**Background:**

Diabetic retinopathy (DR) remains one of the leading causes of visual impairment worldwide. Recent studies have suggested that hemoglobin (Hb) levels may be associated with the risk of DR; however, the evidence has been inconsistent. This study aimed to evaluate the relationship between Hb concentration and the presence of DR among Chinese individuals with type 2 diabetes mellitus (T2DM).

**Methods:**

We performed a cross-sectional analysis including 9, 215 patients with T2DM. Hemoglobin levels were categorized into sex-specific tertiles to examine their association with DR. Multivariable logistic regression models were constructed with adjustment for demographic, behavioral, and clinical covariates. Potential nonlinear relationships were explored using restricted cubic spline (RCS) models. Subgroup and sensitivity analyzes were also conducted to assess the robustness of the findings.

**Results:**

Among all participants, 1, 757 (19.1%) were diagnosed with DR. The prevalence of DR was significantly higher in the lowest Hb tertile compared with the middle and highest tertiles (24.5% vs. 16.4% vs. 16.6%, P < 0.001). After adjusting for potential confounders, each 1 g/L increase in Hb was associated with a 1% reduction in DR risk (adjusted OR 0.99, 95% CI 0.98-0.99). Compared with the lowest tertile, participants in higher Hb tertiles had a lower likelihood of DR (T2: OR 0.68, 95% CI 0.59-0.78, P < 0.001; T3: OR 0.67, 95% CI 0.58-0.77, P < 0.001). These associations were consistent across predefined subgroups (all P for interaction > 0.05) and remained stable in sensitivity analyzes restricted to complete cases. RCS analysis demonstrated a nonlinear, inverted L-shaped association between Hb and DR in men (P for nonlinearity = 0.041), whereas a linear inverse relationship was observed in women (P for nonlinearity = 0.977).

**Conclusions:**

Hb levels are negatively associated with the presence of DR in Chinese patients with T2DM, suggesting that Hb may serve as a useful clinical indicator for DR risk stratification.

## 1 Introduction

Diabetes mellitus is a persistent global public health concern, with type 2 diabetes mellitus (T2DM) accounting for the majority of cases (1). According to estimates from the International Diabetes Federation, more than 537 million adults were living with diabetes in 2021, with a projected increase to 783 million by 2045 (2). China bears the largest burden worldwide, with over 118 million individuals affected, accounting for an estimated 22% of global cases (3). As the prevalence of diabetes continues to increase, the incidence of chronic complications is also expected to rise. Among these, diabetic retinopathy (DR) is a principal cause of preventable blindness globally (4). The pathogenesis of DR is multifactorial, involving chronic hyperglycemia, inflammatory responses, oxidative stress, and microvascular injury, ultimately leading to retinal hypoxia and pathological neovascularization (5).

The onset and progression of DR are influenced by multiple well-established factors, including age, glycemic control, and duration of diabetes (6). However, substantial inter-individual variability exists, even among patients with comparable glycemic profiles, indicating that additional determinants may contribute to DR susceptibility (7). Hemoglobin (Hb), as the primary carrier of oxygen in the bloodstream, plays a critical role in maintaining tissue oxygenation (8). Reduced Hb levels, as seen in anemia, are common in patients with T2DM (9, 10), often in association with chronic kidney disease or systemic inflammation (11, 12). While prior research has largely focused on anemia as a categorical condition, emerging evidence suggests that variations in Hb levels across the full physiological range may also influence the risk of diabetic complications (13, 14). From a mechanistic perspective, lower Hb concentrations may aggravate retinal hypoxia, a central driver of DR development (15). As such, evaluating Hb as a continuous variable may provide a more nuanced understanding of its relationship with DR risk.

Previous epidemiological studies investigating the association between anemia and DR have yielded conflicting results (11, 12, 16–18). Some cross-sectional analyzes have reported an inverse relationship, whereas certain longitudinal studies have suggested that elevated Hb levels may increase the risk of proliferative DR. These discrepancies may reflect differences in study design, population characteristics, sample size, or inadequate adjustment for confounding factors such as sex and renal function. Moreover, limited research has explored potential nonlinear associations or sex-specific differences in this relationship, leaving an important gap in the literature. Accordingly, the present study aimed to investigate the association between Hb levels and the presence of diabetic retinopathy in a large cohort of Chinese patients with T2DM, with particular attention to potential nonlinear patterns and sex-specific effects.

## 2 Materials and methods

### 2.1 Study design and setting

This cross-sectional study utilized data obtained from the National Metabolic Management Center (MMC) at Yuhuan Second People**’**s Hospital. The MMC operates under a standardized framework described as **“**One Center, One Stop, and One Standard Model**, ”** aimed at optimizing the management of metabolic diseases, including T2DM. At enrollment, trained personnel collected patient data according to a unified protocol. Participants underwent baseline assessments and were subsequently scheduled for routine follow-up in accordance with MMC guidelines. Individualized treatment plans were developed based on patient characteristics, and management adhered strictly to current Chinese clinical guidelines for T2DM.

### 2.2 Study participants

**Figure 1:**
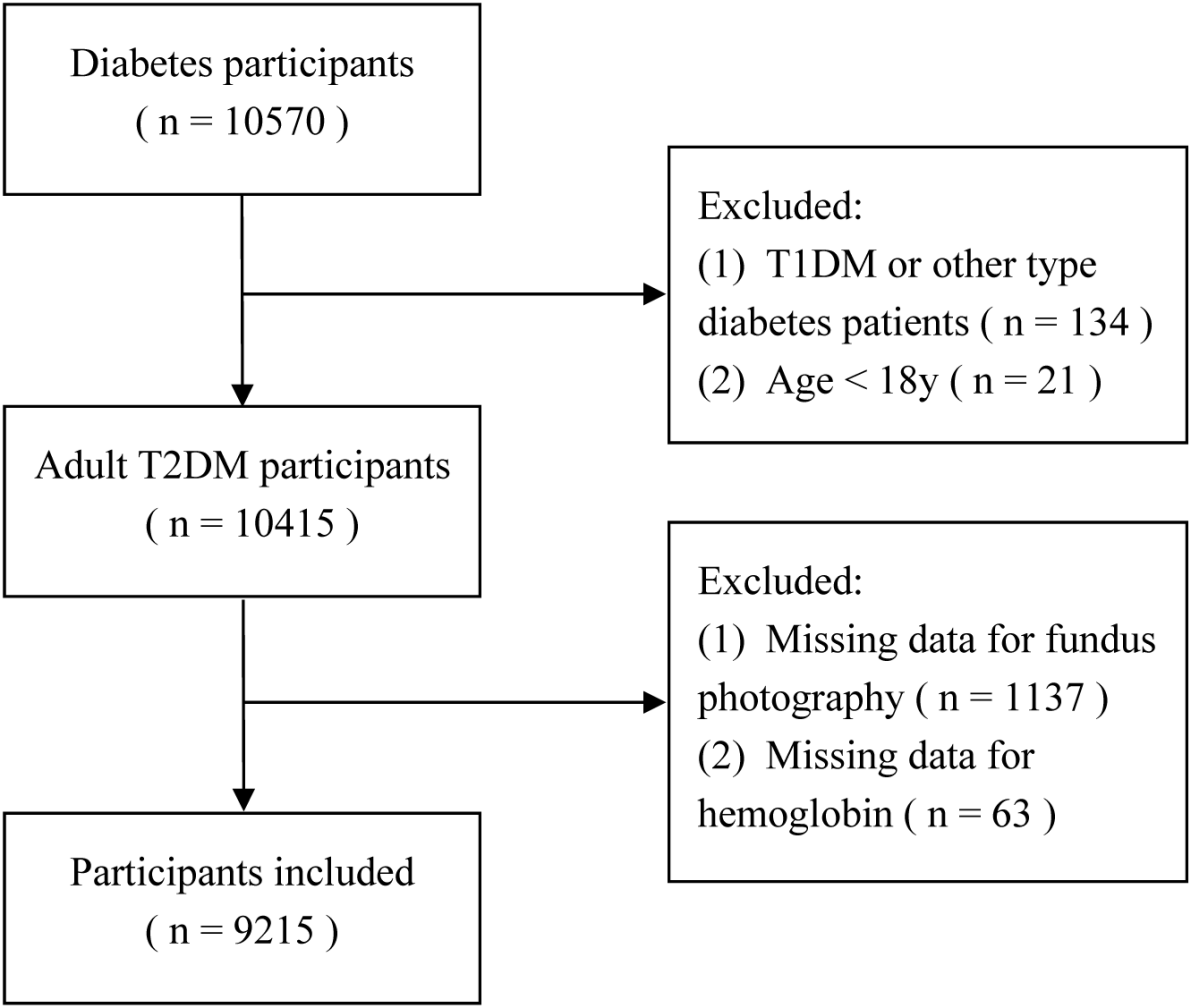
Study flow chart.

Data were extracted from the MMC database for individuals enrolled between September 2017 and February 2025. Of 10, 570 patients with diabetes initially identified, 9, 215 were included in the final analysis. Exclusion criteria comprised: diagnosis of type 1 diabetes or other specific diabetes types (n = 134), age under 18 years (n = 21), and missing data for fundus photography (n = 1, 137) or Hb measurements (n = 63). A flowchart detailing participant selection is presented in **Figure 1**.

The study protocol was approved by the institutional ethics committee (Approval No. 1708) and conducted in accordance with the Declaration of Helsinki. Written informed consent was obtained from all participants at enrollment.

### 2.3 Data collection

At baseline, comprehensive clinical and laboratory data were obtained from the MMC database. Variables included demographic information (sex, age, education level), clinical characteristics (duration of diabetes, hypertension, dyslipidemia), lifestyle factors (smoking and alcohol consumption), anthropometric measures (height, weight), and laboratory parameters. These parameters included systolic and diastolic blood pressure (SBP, DBP), Hb, fasting blood glucose (FBG), fasting C-peptide (FCp), glycated Hb (HbA1c), blood urea nitrogen (BUN), serum creatinine (Scr), uric acid (UA), total cholesterol (TC), triglycerides (TG), high-density lipoprotein cholesterol (HDL-C), and low-density lipoprotein cholesterol (LDL-C). Assessment of DR followed a two-stage procedure incorporating both automated and expert evaluation. Initially, fundus images were analyzed using a deep learning-based system that classified images as gradable (with DR staging) or ungradable due to poor quality (19). Subsequently, all images were independently reviewed by a trained ophthalmologist to confirm or revise the automated classifications. Images deemed ungradable after expert review were excluded. Body mass index (BMI) was calculated as weight (kg) divided by height squared (m²). Estimated glomerular filtration rate (eGFR) was calculated using the Chronic Kidney Disease Epidemiology Collaboration equation (20). Hypertension was defined as SBP ≥ 140 mmHg, DBP ≥ 90 mmHg, or a prior diagnosis with recent use of antihypertensive medication (21). Dyslipidemia was defined based on standard lipid thresholds, including elevated TC, TG, LDL-C, or reduced HDL-C (total cholesterol ≥ 5.7 mmol/L, triglycerides ≥ 1.7 mmol/L, LDL-C ≥ 3.6 mmol/L, or HDL-C < 1.29 mmol/L for women and < 1.03 mmol/L for men) (22). Participants were classified as smokers or drinkers if they reported regular use (daily or near-daily for smoking, weekly or near-weekly for alcohol). Educational attainment was categorized as below high school or high school and above.

### 2.4 Statistical analyzes

Participant characteristics were summarized according to sex-specific tertiles of Hb. Continuous variables were expressed as mean ± standard deviation (SD) for normally distributed data or median with interquartile range (IQR) for non-normally distributed data. Categorical variables were reported as counts and percentages. Differences across Hb tertiles were evaluated using one-way ANOVAs when normally distributed, Kruskal-Wallis H tests when skewed, and chi-square tests for categorical variables. Missing values were addressed using multiple imputation, and detailed information regarding missing covariates is provided in **Supplementary Table 1**. The association between Hb levels and DR was assessed using multivariable binary logistic regression models, with results presented as odds ratios (ORs) and 95% confidence intervals (CIs). Hb was analyzed both as a continuous variable and as a categorical variable based on tertiles. Four progressively adjusted models were constructed. Model 1 included adjustment for sex in continuous analyzes, while no adjustment was required for sex-specific tertile analyzes. Model 2 further adjusted for age. Model 3 additionally incorporated education level, duration of diabetes, smoking status, and alcohol consumption. Model 4 included further adjustment for body mass index (BMI), hypertension, dyslipidemia, fasting C-peptide (FCp), HbA1c, and eGFR. To evaluate potential nonlinear associations between Hb and DR, restricted cubic spline (RCS) regression was applied with adjustment based on Model 4. Nonlinearity was assessed using likelihood ratio tests comparing models with and without spline terms. When a nonlinear relationship was identified, a two-piecewise logistic regression model with smoothing was used to determine threshold effects, with adjustment based on Model 3. Potential effect modification was examined through subgroup analyzes stratified by variables including HbA1c and BMI. Interactions between Hb and subgroup variables were evaluated using likelihood ratio tests. Sensitivity analyzes were conducted using complete-case datasets to assess the impact of missing data imputation. All statistical analyzes were performed using R (v 4.2.2; http://www.R-project.org, The R Foundation). A two-sided P < 0.05 was deemed significant.

## 3 Results

### 3.1 Baseline participant characteristics

A total of 9, 215 participants were included in the final analysis. Baseline characteristics are summarized in **Table 1**. The mean age of the study population was 55.6 ± 11.6 years, and 57.3% of participants were male. Mean Hb levels were notably higher in men than in women (150.6 ± 16.5 g/L vs. 131.5 ± 13.7 g/L). Overall, 1, 757 individuals (19.1%) were diagnosed with DR. When stratified by sex-specific Hb tertiles, the prevalence of DR showed a significant decreasing trend across increasing Hb levels, declining from 24.5% in the lowest tertile to 16.6% in the highest tertile (P < 0.001).

**Table 1:** Baseline participant characteristics.

Variables	Total (n = 9215)	Hb sex‐stratified tertiles			P value
		T1 (n = 2918) male < 146 female < 127	T2 (n = 3095) male ≥ 146, < 158 female ≥ 127, < 138	T3 (n = 3202) male ≥ 158 female ≥ 138	
Male, n (%)	5277 (57.3)	1698 (58.2)	1733 (56)	1846 (57.7)	0.196
Age, y	55.6 ± 11.6	59.9 ± 11.4	55.3 ± 10.8	51.9 ± 11.2	< 0.001
High school education and above, n (%)	888 (9.6)	184 (6.3)	313 (10.1)	391 (12.2)	< 0.001
DBP, mmHg	75.1 ± 11.3	71.6 ± 11.3	75.2 ± 10.7	78.3 ± 10.9	< 0.001
SBP, mmHg	131.6 ± 18.4	130.4 ± 19.5	131.5 ± 18.0	132.9 ± 17.6	< 0.001
BMI, kg/m2	25.6 ± 3.7	25.0 ± 3.6	25.8 ± 3.6	26.1 ± 3.7	< 0.001
Duration of diabetes, y	2.8 (0.1, 9.4)	5.0 (1.0, 11.5)	2.6 (0.1, 8.5)	1.4 (0.0, 5.6)	< 0.001
Hypertension, n (%)	5327 (57.8)	1843 (63.2)	1724 (55.7)	1760 (55)	< 0.001
Dyslipidemia, n (%)	6152 (66.8)	1674 (57.4)	2049 (66.2)	2429 (75.9)	< 0.001
Smoking, n (%)	2304 (25.0)	648 (22.2)	707 (22.8)	949 (29.6)	< 0.001
Alcohol consumption, n (%)	1215 (13.2)	300 (10.3)	442 (14.3)	473 (14.8)	< 0.001
FBG, mmol/L	9.3 ± 4.2	8.5 ± 3.8	9.0 ± 3.7	10.4 ± 4.6	< 0.001
FCp, ng/mL	2.3 (1.7, 3.0)	2.1 (1.5, 2.9)	2.2 (1.7, 2.9)	2.5 (1.8, 3.2)	< 0.001
HbA1c, %	8.5 ± 2.2	8.1 ± 2.2	8.3 ± 2.1	8.9 ± 2.3	< 0.001
BUN, mmol/L	5.4 (4.4, 6.5)	5.8 (4.6, 7.2)	5.3 (4.4, 6.3)	5.2 (4.3, 6.1)	< 0.001
Scr, μmol/L	62.0 (51.0, 75.0)	68.0 (54.2, 86.0)	60.0 (50.0, 73.0)	59.0 (49.0, 70.0)	< 0.001
eGFR, mL/min per 1.73 m^2^	105.1 (85.4, 127.6)	93.5 (71.6, 115.6)	106.4 (89.4, 127.8)	112.7 (94.2, 134.5)	< 0.001
UA, μmol/L	337.8 ± 100.2	348.3 ± 109.8	333.3 ± 93.2	332.8 ± 96.7	< 0.001
TG, mmol/L	1.5 (1.0, 2.3)	1.3 (0.9, 2.0)	1.5 (1.0, 2.2)	1.7 (1.2, 2.6)	< 0.001
TC, mmol/L	5.3 ± 1.3	4.9 ± 1.3	5.3 ± 1.2	5.6 ± 1.4	< 0.001
HDL-C, mmol/L	1.2 ± 0.3	1.1 ± 0.3	1.2 ± 0.3	1.2 ± 0.3	< 0.001
LDL-C, mmol/L	3.0 ± 1.0	2.7 ± 1.0	3.0 ± 1.0	3.2 ± 1.0	< 0.001
Hb, g/L	142.4 ± 18.1	125.4 ± 14.6	143.2 ± 10.2	157.2 ± 12.6	< 0.001
Male	150.6 ± 16.5	132.3 ± 13.3	151.8 ± 3.3	166.4 ± 7.2	< 0.001
Female	131.5 ± 13.7	115.8 ± 10.4	132.2 ± 3.2	144.8 ± 6.0	< 0.001
DR, n (%)	1757 (19.1)	715 (24.5)	509 (16.4)	533 (16.6)	< 0.001

Hb tertiles based on separate tertile intervals for males and females in g/L.Hb, hemoglobin; T, tertile; DBP, diastolic blood pressure; SBP, systolic blood pressure; BMI, body mass index; FBG, fasting blood glucose; FCp, fasting serum C peptide; HbA1c, glycated hemoglobin; BUN, urea nitrogen; Scr, serum creatinine; eGFR, estimated glomerular filtration rate; UA, uric acid; TG, triglycerides; TC, total cholesterol; HDL-C, high-density lipoprotein cholesterol; LDL-C, low-density lipoprotein cholesterol; DR, diabetic retinopathy.

### 3.2 Relationship between Hb and DR in T2DM

Multivariable logistic regression analyzes demonstrated a significant inverse association between Hb levels and the presence of DR. When Hb was analyzed as a continuous variable, higher Hb levels were associated with a lower likelihood of DR after adjustment for sex (OR 0.98, 95% CI 0.98-0.99, P < 0.001; **Table 2**). This relationship remained statistically significant after full adjustment for potential confounders (OR 0.99, 95% CI 0.98-0.99, P < 0.001; **Table 2**). When Hb was categorized into sex-specific tertiles, participants in higher tertiles exhibited significantly reduced odds of DR compared with those in the lowest tertile. Specifically, the adjusted ORs were 0.68 (95% CI 0.59-0.78, P < 0.001) for the second tertile and 0.67 (95% CI 0.58-0.77, P < 0.001) for the third tertile, after controlling for all covariates (**Table 2**).

**Table 2:** Multivariate logistic regression analysis of the association between hemoglobin and diabetic retinopathy in type 2 diabetes mellitus.

Variable	Model 1		Model 2		Model 3		Model 4	
	OR (95% CI)	P value	OR (95% CI)	P value	OR (95% CI)	P value	OR (95% CI)	P value
Hb, g/L	0.98 (0.98~0.99)	<0.001	0.99 (0.98~0.99)	<0.001	0.99 (0.99~0.99)	<0.001	0.99 (0.98~0.99)	<0.001
Hb sex‐stratified tertiles								
T1	1(Ref)		1(Ref)		1(Ref)		1(Ref)	
T2	0.61 (0.53~0.69)	<0.001	0.61 (0.54~0.7)	<0.001	0.67 (0.59~0.77)	<0.001	0.68 (0.59~0.78)	<0.001
T3	0.62 (0.54~0.7)	<0.001	0.63 (0.55~0.72)	<0.001	0.73 (0.64~0.84)	<0.001	0.67 (0.58~0.77)	<0.001

Model 1, adjusted for gender in continuous analyzes, no adjustment for sex-adjusted tertiles; Model 2, adjusted as for model 1, additionally adjusted for age; Model 3, adjusted as for model 2, additionally adjusted for education level, duration of diabetes, smoking, alcohol consumption; Model 4, adjusted as for model 3, additionally adjusted for BMI, hypertension, dyslipidemia, FCp, HbA1c, eGFR.

**Figure 2:**
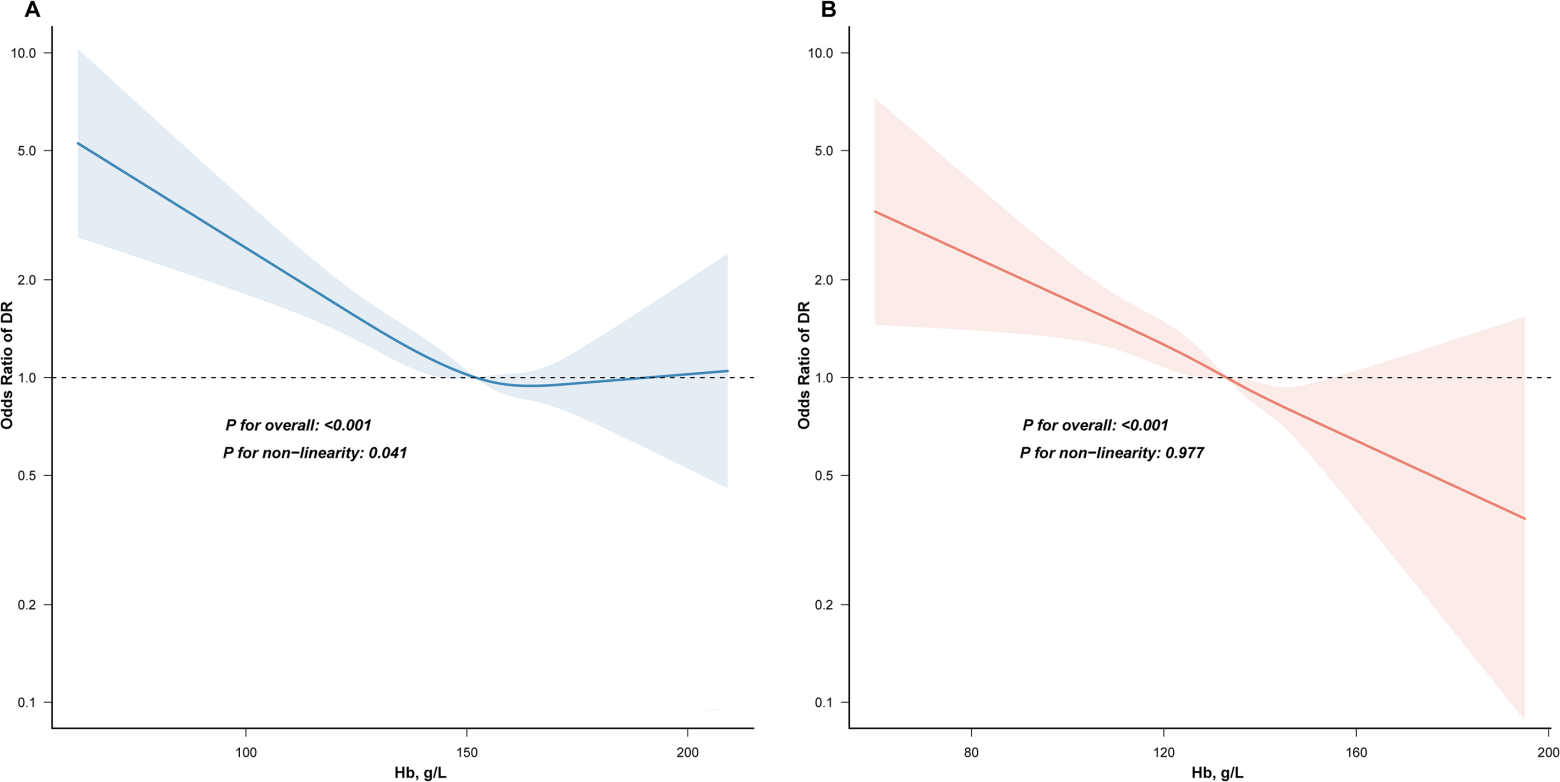
Association between hemoglobin levels and diabetic retinopathy in male **(A)** and female **(B)** participants with T2DM. Models were adjusted for age, education level, duration of diabetes, smoking, alcohol consumption, BMI, hypertension, dyslipidemia, FCp, HbA1c, and eGFR. The red curve represents the fitted relationship between hemoglobin and the odds ratio of DR, with shaded areas indicating 95% confidence intervals. The blue histogram illustrates the distribution of hemoglobin values in the study population.

Given the established sex differences in Hb levels, sex-stratified RCS analyzes were performed (**Figure 2**). In men, the relationship between Hb and DR followed a nonlinear, inverted L-shaped pattern (P for nonlinearity = 0.041; **Figure 2A**). Threshold analysis identified a cutoff at 151.337 g/L. Below this value, each 1 g/L increase in Hb was associated with a 2% reduction in DR risk (OR 0.980, 95% CI 0.973-0.987, P < 0.001; **Table 3**). Above this threshold, no significant association was observed, suggesting a plateau effect. In contrast, among female participants, Hb levels demonstrated a linear negative relationship with DR risk (P for nonlinearity = 0.977; **Figure 2B**).

**Table 3:** Threshold effect analysis of the association between hemoglobin and diabetic retinopathy in males with type 2 diabetes.

Hb, g/L	Adjusted model	
	OR (95% CI)	P-value
< 151.337	0.980 (0.973~0.987)	< 0.001
≥ 151.337	1.005 (0.992~1.018)	0.457
Likelihood Ratio test		0.016

Hb, hemoglobin; OR, odds ratio; CI, confidence interval. Adjusted for age, education level, duration of diabetes, smoking, alcohol consumption, BMI, hypertension, dyslipidemia, FCp, HbA1c, eGFR.

### 3.3 Subgroup analysis

**Figure 3:**
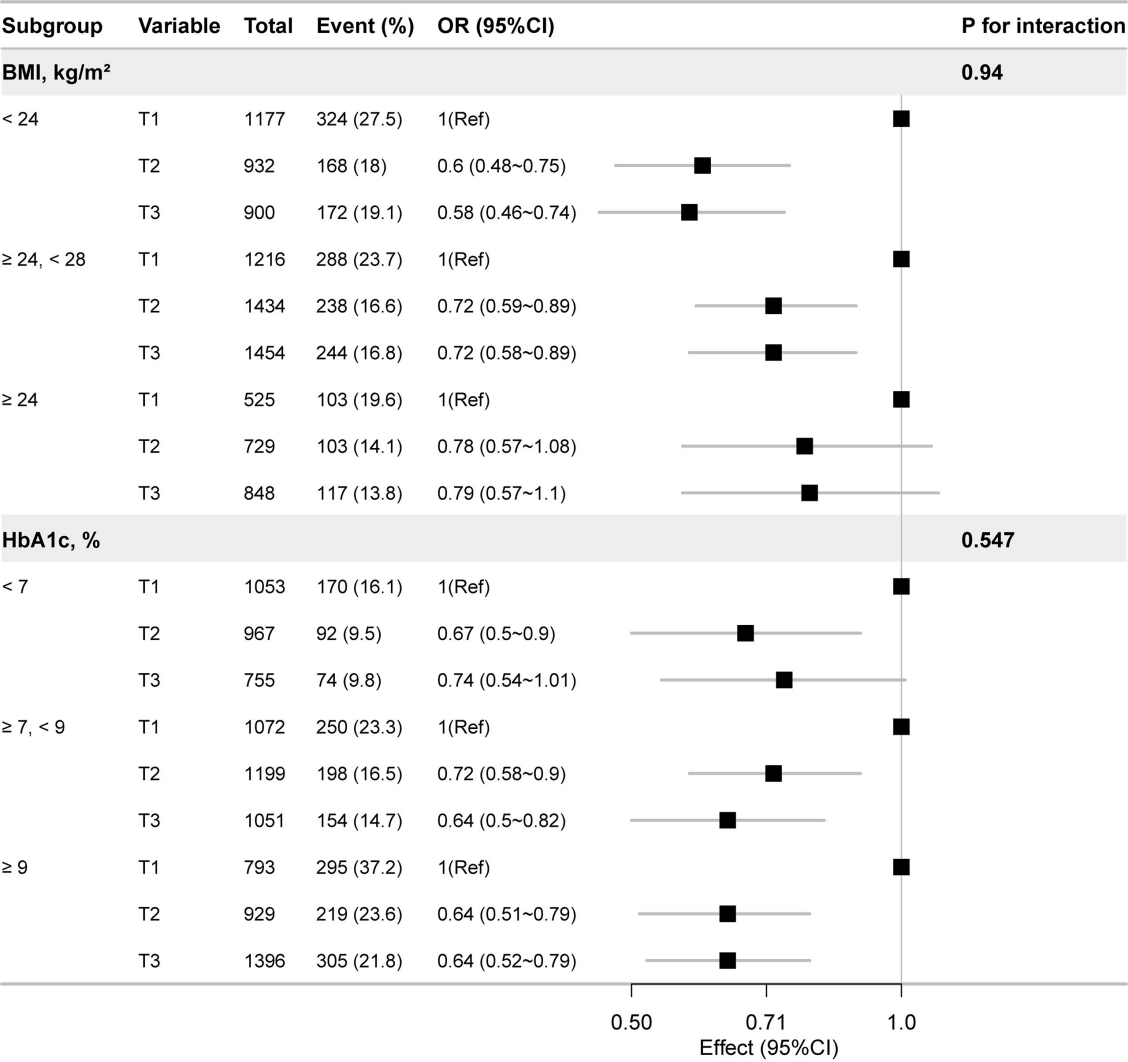
Subgroup analyzes of the association between hemoglobin and diabetic retinopathy in patients with T2DM. All variables were adjusted for the same covariates as above, except for the stratification variable itself.

Stratified analyzes were conducted to explore potential effect modification by key variables. The inverse association between Hb and DR remained consistent across subgroups defined by BMI and HbA1c levels. No statistically significant interactions were identified (all P for interaction > 0.05), indicating that the relationship between Hb and DR was stable across these subpopulations (**Figure 3**).

### 3.4 Sensitivity analysis

Sensitivity analyzes were performed to evaluate the robustness of the primary findings. Results derived from complete-case analyzes were consistent with those obtained after multiple imputation (**Supplementary Table 2**). Following full adjustment, Hb remained inversely associated with DR (OR 0.99, 95% CI 0.98-0.99, P < 0.001). Similarly, higher Hb tertiles continued to demonstrate a significantly lower risk of DR compared with the lowest tertile (T2: OR 0.68, 95% CI 0.60-0.78, P < 0.001; T3: OR 0.68, 95% CI 0.59-0.78, P < 0.001).

## 4 Discussion

In this large cross-sectional study of 9, 215 individuals with T2DM, we systematically evaluated the association between Hb levels and diabetic retinopathy using sex-specific Hb stratification. Our findings indicate a robust inverse relationship between Hb concentration and DR prevalence. Even after adjusting for established risk factors including age, glycemic control (HbA1c), duration of diabetes, hypertension, and renal function (eGFR), lower Hb levels remained independently associated with increased odds of DR. Moreover, sex-specific patterns for this association were detected through RCS analysis. In men, the relationship followed a nonlinear, inverted L-shaped curve, with a threshold identified at 151.337 g/L. Below this level, increasing Hb was associated with a progressively lower risk of DR, whereas no additional benefit was observed beyond the threshold. In contrast, women exhibited a linear inverse association across the full range of Hb values.

Our results are consistent with prior cross-sectional studies conducted in diverse populations. For example, a study of 2, 123 Korean patients with T2DM reported a significant inverse association between Hb levels and DR risk, with each 1.0 g/dL increase in Hb corresponding to a 19% reduction in risk (16). Similarly, analyzes of NHANES data demonstrated a negative correlation between Hb and DR after multivariable adjustment (14). Consistently, Yin et al. (13) also reported an 18% reduction in DR prevalence per 1 g/dL increase in Hb among Tibetan men. Importantly, the present study extends previous work by incorporating sex-specific Hb tertiles, thereby providing a more precise adjustment for physiological differences between men and women. In addition, the identification of a nonlinear association with a defined threshold in men offers novel insight into the dose-response relationship between Hb and DR. In contrast, some studies have reported inconsistent findings. For instance, Traveset et al. (17) observed an inverse association between Hb and DR severity only in unadjusted analyzes, with the relationship becoming nonsignificant after multivariable adjustment. This discrepancy may be attributable to their relatively small sample size. Furthermore, a longitudinal study by Conway et al. (18) involving patients with type 1 diabetes reported a positive association between Hb levels and the risk of proliferative DR, particularly in men. Differences in diabetes type, disease duration, and underlying pathophysiology may account for these divergent observations.

Several biological mechanisms may underlie the observed association between Hb levels and diabetic retinopathy. First, Hb plays a central role in oxygen delivery, and reduced levels may contribute to retinal hypoxia (23). Hypoxic conditions can activate hypoxia-inducible factors, which in turn stimulate vascular endothelial growth factor (VEGF) expression, promoting pathological neovascularization and increased vascular permeability, both of which are central to the progression of DR (24, 25). In addition, decreased Hb or anemia may induce compensatory hemodynamic responses (26), including increased retinal blood flow and elevated glomerular filtration. These changes may enhance endothelial shear stress and accelerate microvascular injury in the retina, paralleling mechanisms described in diabetic nephropathy (27, 28). Iron metabolism provides another plausible link. Although iron is essential for physiological function, it can also facilitate the generation of reactive oxygen species (ROS) through the Fenton reaction (29). Lower Hb levels, particularly in the setting of anemia of chronic disease, as are frequently observed in diabetes, may reflect disturbances in iron homeostasis and contribute to heightened oxidative stress (30, 31). This pro-oxidative environment can damage retinal endothelial cells and pericytes, promote the accumulation of advanced glycation end-products, and further compromise vascular integrity (32). The nonlinear pattern identified in male participants, characterized by a threshold effect, suggests a more complex relationship in which the impact of Hb may vary across different concentration ranges. This phenomenon could be influenced by sex-specific biological factors, such as hormonal regulation or other unmeasured variables (33, 34).

From a clinical perspective, these findings have several important implications for the management of T2DM. Hb, as a routinely measured and widely accessible laboratory parameter, may serve as an additional biomarker for identifying patients at increased risk of developing DR, complementing established risk indicators. These results also highlight the importance of recognizing and managing anemia in individuals with diabetes, not only to improve overall health but also potentially to mitigate ocular complications. At the population level, the findings support consideration of incorporating hematologic indicators into strategies for preventing diabetic complications. This study also has several methodological strengths. The large sample size of 9, 215 participants drawn from a standardized metabolic management framework enhances statistical power and supports the applicability of the findings within the T2DM population. The analytical strategy was comprehensive, incorporating both continuous and categorical representations of Hb, including sex-specific tertiles to account for physiological differences. Furthermore, the use of restricted cubic spline modeling enabled the identification of a nuanced nonlinear association in men, extending beyond conventional linear assumptions. The consistency of results through multiple sensitivity analyzes, including complete-case evaluation, further reinforces the robustness of the findings. In addition, extensive adjustment for potential confounders, including glycemic control, renal function, and disease duration, strengthens the evidence for an independent relationship linking Hb levels and DR.

Despite these strengths, several limitations should be acknowledged. This was a single-center study based on the Taizhou MMC cohort, which may limit the generalizability of the findings to other clinical settings or populations; external validation in multicenter studies is therefore warranted. The study population consisted exclusively of Chinese individuals with T2DM, and extrapolation to other ethnic groups should be approached with caution, as genetic and lifestyle factors may influence the observed associations. The cross-sectional design precludes causal inference, limiting interpretation to associations rather than temporal relationships. Additionally, although a broad range of confounders was considered, residual or unmeasured confounding such as dietary patterns, medication use, or other biological markers cannot be entirely excluded. Future research should include prospective cohort studies to clarify causality and temporal dynamics, as well as interventional trials to determine whether correction of anemia or optimization of Hb levels within physiological ranges can reduce the risk or progression of DR.

## 5 Conclusions

In this cross-sectional analysis of Chinese patients with T2DM, Hb levels were inversely associated with the presence of diabetic retinopathy. This relationship persisted after adjustment for multiple confounding factors and demonstrated a nonlinear, threshold-dependent relationship among male patients. These results suggest that Hb may represent a practical and informative biomarker for evaluating DR risk in clinical settings.

## Data Availability

The raw data supporting the conclusions of this article will be made available by the authors, without undue reservation.
