# Supplementary material for: Relationship between hemoglobin levels and diabetic retinopathy in Chinese type 2 diabetes mellitus populations: a cross-sectional study
Source: Front Endocrinol (Lausanne). 2026 May 14;17:1800238. doi: 10.3389/fendo.2026.1800238 (PMC13215896; doi:10.3389/fendo.2026.1800238)
Supplement: Supplementary file 1 [file Table1.docx]

**Supplementary Table 1 Missing data and miss rates of covariates**

| **Variables** | **Nonmissing** | **Missing** | **Missing Rate (%)** |
| --- | --- | --- | --- |
| Gender | 9215 | 0 | 0.0 |
| Age | 9215 | 0 | 0.0 |
| Education level | 9214 | 1 | 0.0 |
| DBP | 9215 | 0 | 0.0 |
| SBP | 9215 | 0 | 0.0 |
| BMI | 9215 | 0 | 0.0 |
| Duration of diabetes | 9215 | 0 | 0.0 |
| Hypertension | 9215 | 0 | 0.0 |
| Dyslipidemia | 9215 | 0 | 0.0 |
| Smoking | 9215 | 0 | 0.0 |
| Alcohol consumption | 9215 | 0 | 0.0 |
| FBG | 9212 | 3 | 0.0 |
| FCp | 9172 | 43 | 0.5 |
| HbA1c | 9197 | 18 | 0.2 |
| BUN | 9187 | 28 | 0.3 |
| Scr | 9189 | 26 | 0.3 |
| e-GFR | 9189 | 26 | 0.3 |
| UA | 9180 | 35 | 0.4 |
| TG | 9195 | 20 | 0.2 |
| TC | 9194 | 21 | 0.2 |
| HDL-C | 9192 | 23 | 0.2 |
| LDL-C | 9194 | 21 | 0.2 |
| Hb | 9215 | 0 | 0.0 |

All abbreviations are listed in Table 1.
