# Supplementary material for: Relationship between hemoglobin levels and diabetic retinopathy in Chinese type 2 diabetes mellitus populations: a cross-sectional study
Source: Front Endocrinol (Lausanne). 2026 May 14;17:1800238. doi: 10.3389/fendo.2026.1800238 (PMC13215896; doi:10.3389/fendo.2026.1800238)
Supplement: Supplementary file 2 [file Table2.docx]

**Supplementary Table 2 Association between hemoglobin and diabetic retinopathy in patients with type 2 diabetes**

| Variable | Model 1 | | Model 2 | | Model 3 | | Model 4 | |
| --- | --- | --- | --- | --- | --- | --- | --- | --- |
|  | OR (95%CI) | P value | OR (95%CI) | P value | OR (95%CI) | P value | OR (95%CI) | P value |
| Hb | 0.98 (0.98~0.99) | <0.001 | 0.99 (0.98~0.99) | <0.001 | 0.99 (0.99~0.99) | <0.001 | 0.99 (0.98~0.99) | <0.001 |
| Hb Group |  |  |  |  |  |  |  |  |
| T1 | 1(Ref) |  | 1(Ref) |  | 1(Ref) |  | 1(Ref) |  |
| T2 | 0.61 (0.53~0.69) | <0.001 | 0.61 (0.54~0.7) | <0.001 | 0.68 (0.59~0.77) | <0.001 | 0.68 (0.6~0.78) | <0.001 |
| T3 | 0.62 (0.54~0.7) | <0.001 | 0.63 (0.55~0.72) | <0.001 | 0.73 (0.64~0.84) | <0.001 | 0.68 (0.59~0.78) | <0.001 |

Model 1: adjusted for gender in continuous analyses, no adjustment for sex-adjusted tertiles;

Model 2: adjusted as for model 1, additionally adjusted for age;

Model 3: adjusted as for model 2, additionally adjusted for education level, duration of diabetes, smoking, alcohol consumption;

Model 4: adjusted as for model 3, additionally adjusted for BMI, hypertension, dyslipidemia, FCp, HbA1c, eGFR.
